# Management of familial Mediterranean fever by colchicine does not normalize the altered profile of microbial long chain fatty acids in the human metabolome

**DOI:** 10.3389/fcimb.2013.00002

**Published:** 2013-01-28

**Authors:** Zhanna A. Ktsoyan, Natalia V. Beloborodova, Anahit M. Sedrakyan, George A. Osipov, Zaruhi A. Khachatryan, Gayane P. Manukyan, Karine A. Arakelova, Alvard I. Hovhannisyan, Arsen A. Arakelyan, Karine A. Ghazaryan, Magdalina K. Zakaryan, Rustam I. Aminov

**Affiliations:** ^1^Institute of Molecular Biology, National Academy of SciencesYerevan, Republic of Armenia; ^2^Research Institute of General Reanimatology, Russian Academy of Medical SciencesMoscow, Russian Federation; ^3^Scientific Center of Cardiovascular Surgery, Russian Academy of Medical SciencesMoscow, Russian Federation; ^4^Faculty of Medical Sciences, University of the West IndiesKingston, Jamaica

**Keywords:** familial Mediterranean fever, colchicine, metabolome, gas chromatography/mass spectrometry (GC/MS), microbial long chain fatty acids, discriminant function analysis

## Abstract

In our previous works we established that in an autoinflammatory condition, familial Mediterranean fever (FMF), the gut microbial diversity is specifically restructured, which also results in the altered profiles of microbial long chain fatty acids (LCFAs) present in the systemic metabolome. The mainstream management of the disease is based on oral administration of colchicine to suppress clinical signs and extend remission periods and our aim was to determine whether this therapy normalizes the microbial LCFA profiles in the metabolome as well. Unexpectedly, the treatment does not normalize these profiles. Moreover, it results in the formation of new distinct microbial LCFA clusters, which are well separated from the corresponding values in healthy controls and FMF patients without the therapy. We hypothesize that the therapy alters the proinflammatory network specific for the disease, with the concomitant changes in gut microbiota and the corresponding microbial LCFAs in the metabolome.

## Introduction

The gut microbial symbionts became intimately integrated into the normal metabolism and physiology of human beings during the long-term co-evolutionary history of host-microbe interaction (Bäckhed et al., 2005; Ley et al., 2006). The gut microbiota evolved to play an important role in many aspects of human health and development, including the development and proper functioning of the immune system (Macpherson and Harris, 2004; Artis, 2008; Mazmanian et al., 2008). The optimal host-microbiota balance that has been achieved during the extended co-evolutionary process shaped a particular set of interactions that is dependent on a number of environmental and genetic factors (Spor et al., 2011).

Unlike other animals, however, the lifestyle of humans changed dramatically due to the development of agriculture and urban living. This cardinally changed the living conditions compared to those of our ancestors, which now include a highly hygienic living environment, refined, and high calorie diets, infectious disease control through vaccination and use of antibiotics, and many other achievements of our civilization. Due to a more protected and resourceful lifestyle and advances in medicine that allowed controlling many infectious and hereditary diseases, the humans also managed to escape the stabilizing pressure of natural selection. Moreover, genetics of certain complex diseases such as Type 1 Diabetes or Rheumatoid Arthritis shows the signs of a recent positive selection for SNPs associated with the disease susceptibility (Corona et al., 2010).

Thus the fine-tuned host-microbiota interaction, which has been built and refined during hundreds of millennia, in certain cases in contemporary life may be compromised and result in severe disease. A very good example of this dysfunctional interaction between the host and commensal microbiota is inflammatory bowel disease (IBD), which involves both the environmental and genetic components (Sartor, 2006; Van Limbergen et al., 2007). Besides the clinical manifestations, a broader picture of the disease includes a heavily biased structure of gut microbiota (Manichanh et al., 2006; Frank et al., 2007; Qin et al., 2010).

In our previous works we focused on host-microbiota interaction in an autoinflammatory disease, familial Mediterranean fever (FMF, MIM249100) (Khachatryan et al., 2008; Manukyan et al., 2008b; Ktsoyan et al., 2010). This disease is the most common member of hereditary autoinflammatory diseases and it is characterized by recurrent self-resolving acute attacks of fever and polyserositis. Mutations responsible for the disease onset and periodic inflammation are located in the *MEFV* gene (The French FMF Consortium, 1997; The International FMF Consortium, 1997), which encodes the protein called pyrin or marenostrin, one of the regulators of innate immunity.

The mutations in the gene are ancient and the estimated ages for the most recent common ancestor for the frequently encountered mutations such as M694V, M694I, V726A, M680I, and E148Q are 7000, 8500, 15,000, 23,000, and 30,000 years, respectively (Jalkh et al., 2008). Jews are the most probable founder population for several common MEFV mutations (Papadopoulos et al., 2008). This is most likely due to early genetic isolation and small population sizes at times (bottleneck effect), the prerequisites for intensive genetic drift and mutation fixation. The mutations, however, penetrated into other populations originating from the Mediterranean basin (Armenians, Arabs, and Turks) and the carrier frequency in these populations is very high (Aksentijevich et al., 1999). These frequencies cannot be explained solely by intense inbreeding and increased genetic drift though. Moreover, the presence of recurrent mutations in *MEFV*, with the reappearance of an ancestral amino acid state, implies a strong positive selection (Schaner et al., 2001). Interestingly, amino acid substitutions that cause human disease are often present as wild type in other species of primates suggesting that the effect of mutations is dependent on the environmental/genetic context. Still, it remains an open question, what kind of selective advantage had been conferred by these *MEFV* alleles to the prehistoric human populations as well as to the primates in the wild nowadays. In the contemporary living environment of humans, the mutation effects are debilitating and result in the increased morbidity and mortality if left untreated.

Clinically, the disease is characterized by acute self-resolving attacks of fever with serositis such as peritonitis, pleuritis, and arthritis, with a massive influx of polymorphonuclear leukocytes into the affected tissues. In remission the patients are clinically asymptomatic although a number of inflammation markers are elevated suggesting a persisting subclinical inflammation (Manukyan et al., 2008a). Factors contributing to the recurrent disease attacks remain elusive and thought to be due to emotional, physiological, or physical stress, change of diet, menstrual periods, and other, less defined factors (Sohar et al., 1967; Schwabe and Peters, 1974). In a recent study employing the case-crossover design, the authors found a positive, statistically significant relation between the number of stressful events and FMF attacks (Yenokyan and Armenian, 2012). The results of this epidemiological study are consistent with the earlier intervention studies that induced FMF-resembling attacks in FMF patients, but not in control subjects, by infusion of metaraminol, a potent sympathomimetic amine used in the prevention and treatment of hypotension (Barakat et al., 1984). It is not an improper sympatho-adrenal response to stress though because the responses to metaraminol as well as to stressful conditions in the form of attacks are delayed (Barakat et al., 1984; Yenokyan and Armenian, 2012). Most likely, the catecholamines induce the secondary effect such as the increased exposure to gut bacterial products due to the decreased gut motility. Interestingly, the metaraminol-induced attacks can be prevented with prophylactic colchicine therapy (Barakat et al., 1984), which serves as another evidence to rule out the direct involvement of the hypothalamic-pituitary-adrenal axis response in disease attacks.

The mainstream management of the disease is based on the daily *per os* administration of colchicine, which has a proven record of prevention of attacks and amyloid deposition in FMF (Kallinich et al., 2007). This antimitotic drug helps to reduce the frequency, duration and intensity of disease relapses and extend the remission periods. It is effective in prevention of FMF complications such as amyloidosis and renal failure too (Niel and Scherrmann, 2006). Colchicine is also used for the management of Behcet's disease, gout and cirrhosis and it is difficult to propose the unifying mechanistic explanation for the positive effect of this drug on the diseases with such different etiologies (Ben-Chetrit et al., 2006).

Previously, we showed that this autoinflammatory condition results in cardinal restructuring of gut microbiota (Khachatryan et al., 2008), accompanied by changes in microbial products circulating in the systemic metabolome (Ktsoyan et al., 2010). We also demonstrated the increased systemic reactivity against the commensal gut microbiota in FMF, thus suggesting a possible mechanism for restructuring the gut microbiota composition in an autoinflammatory condition (Manukyan et al., 2008b). In the present work our main aim was to investigate if the colchicine therapy, which is used to suppress the disease manifestations and extend remission periods, is also “normalizing” the metabolome composition, in particular its microbial compound concentrations and profiles.

## Materials and methods

### Subjects

A total of 34 FMF patients (11 females and 23 males, aged from 15 to 54 years) and 43 healthy subjects (23 females and 20 males, aged from 25 to 42 years) were enrolled in this study. In the FMF group, the cohorts comprised of: patients in attack period (1) without colchicine treatment (*n* = 9) and (2) colchicine-treated (*n* = 13); patients in remission period (3) without colchicine treatment (*n* = 5) and (4) colchicine-treated (*n* = 7). All cases of FMF were diagnosed based on Tel-Hashomer criteria (Livneh et al., 1997) with further genetic confirmation for the carriage of the *MEFV* mutations. Colchicine for management of FMF was administered orally at the daily dose of 0.5–1.0 mg and the drug was taken for at least a year. Patients that administered the drug only during attack periods were excluded from the analysis. The control group comprised of healthy volunteers without any family history of FMF; they were also checked for the *MEFV* mutations. They did not suffer from any disease during the sampling period. None of the FMF patients and healthy individuals had used antibiotics within at least 3 months prior to sampling. All study participants gave their informed consent to the protocol, which was approved by the local ethical committee of the Institute of Molecular Biology, NAS RA.

### Sample preparation

Venous blood samples for gas chromatography/mass spectrometry (GC/MS) analysis were collected into sterile heparinized vials and immediately frozen at −20°C until analysis. Defrosted blood samples (50 μl) were air dried at 80°C and subjected to acid methanolysis using 0.4 ml of 1M HCl in methanol for 1 h at 80°C. As a result, fatty acids and aldehydes were extracted from the complex microbial lipids in the form of methyl esters and aldehyde dimethyl acetals. Internal standard, deuteromethyl ether of tridecane acid, prepared in hexane, was added to the cooled reaction mixtures at 300 ng. The fraction of methyl esters of fatty acids with other lipid components was extracted twice from the reaction mixture with 200 μl of hexane. The hexane extracts were dried at 40°C, and the dry residue was treated with 20 μl of N,O-bis-(trimethylsilyl)trifluoroacetamide for 15 min at 80°C to form trimethylsilyl derivatives of hydroxy acids (Beloborodova and Osipov, 2000).

### GC/MS analysis

A GC/MS system (Agilent AT-5973, Agilent Technologies) with a cross-linked methyl silicone capillary column (DB-5ms EVDX; dimensions: 0.2 mm × 25 m × 0.33 mkm, Agilent Technologies) was used to identify microbial components and metabolites in blood. Two microliters of a sample derived as described in the previous section was injected into the capillary column. Fatty acids and other lipid components, separated in the GC column, were analyzed in the selected ion-monitoring (SIM) mode (multi-ion mass-fragmentography regimen). The substances eluted were identified by measuring the specific retention times and the ratio of chromatographic peak areas for selected ions were measured primarily in the automatic mode by using the standard library search program (NIST 02 MS library), with a subsequent manual check of peaks. Correlation between the products identified and the corresponding source microorganisms was carried out as described before (Osipov and Turova, 1997; Osipov et al., 2009) using the Sherlock® Microbial 2 Identification System (MIS) (www.midi-inc.com).

### Statistical analyses

Statistical analyses were performed using the SPSS package (SPSS Inc., Chicago, IL, USA). The Mann–Whitney *U*-test was applied to determine statistical significance among the mean values of studied groups. *P*-values below 0.05 were considered as statistically significant. Discriminant function analysis (DA) was used to classify cases into groups. The investigated groups of subjects were taken as dependent variables of DA and concentrations of microbial components in the blood were taken as predictors.

## Results

We determined the concentrations of systemic hydroxy, branched, cyclopropyl, and unsaturated fatty acids as well as aldehydes among the FMF and control cohorts. The results are presented in the form of median and interquartile range (25th and 75th percentiles) for all five cohorts studied (Table 1). Statistical significance of pairwise differences among the groups is also shown.

**Table 1:** **Concentration of microbial compounds and the potential source in blood of FMF patients and healthy controls**.

**Substance**	**Potential microbial source^a^**	**Healthy controls (*n* = 43)**	**FMF attack**		**FMF remission**	
			**Colchicine − ^d^ (*n* = 9)**	**Colchicine +^e^ (*n* = 13)**	**Colchicine − ^d^ (*n* = 5)**	**Colchicine +^e^ (*n* = 7)**
**HYDROXY FATTY ACIDS (FA)**						
2-hydroxy lauric (2 hC12:0)^b^	*Pseudomonas aeruginosa, Acinetobacter*	0.90 (0.70–1.10)^c^	0.8 (0.6–1.6)	1.4 (1.1–1.9)^*^	1.5 (1.5–3.9)^*^	2.4 (1.75–3.90)^*^^#^
3-hydroxy lauric (3 hC12:0)	*Pseudomonas, Moraxella, Acinetobacter*	2.70 (2.00–3.65)	1.5 (1.1–2.8)	2.8 (1.6–3.5)^†^	4.2 (4.0–4.2)^#^	5.60 (5.00–6.15)^*^^#^
3-hydroxy stearic (3 hC18:0)	*Helicobacter pylori*	11.3 (9.2–16.0)	10.9 (9.9–12.8)	18.5 (10.8–44.0)^*^	40.9 (21.8–56.1)^*^^#^	20.1 (19.0–46.4)^*^^#^
10-hydroxy stearic (10 hC18:0)	*Clostridium perfringens*	27.4 (16.7–36.5)	28.8 (20.4–33.1)	43.4 (27.1–57.5)^*^	64.8 (54.9–67.8)^*^^#^	43.9 (35.4–95.7)^*^^#^
**BRANCHED FA**						
*iso*-myristic (iC14:0)	*Peptostreptococcus, Streptomyces, Bacillus, Bacteroides*,	79.8 (50.7–108.8)	49.4 (40.9–119.6)	61.9 (44.2–84.7)	54.9 (30.0–100.5)	81.7 (64.7–114.8)
*iso*-pentadecanoic (iC15:0)	*Propionibacterium, Bacteroides, Staphylococcus*	366 (292–621)	272 (184–568)	454 (264–508)	429 (195–480)	439 (289–639)
*iso*-palmitic (iC16:0)	*Streptomyces, Bacteroides, Micromonospora*	1193 (862–1563)	996 (817–1669)	1167 (902–1342)	1238 (755–1330)	1425 (1074–1823)
*iso*-heptadecanoic (iC17:0)	*Bacillus, Prevotella, Propionibacterium, Staphylococcus*	2794 (1824–3921)	2381 (1924–3354)	2737 (2120–3709)	2660 (1944–2902)	3749 (2880–4092)
*iso*-stearic (iC18:0)	*Peptostreptococcus, Clostridium difficile, Bifidobacterium*	26.7 (16.75–36.5)	26.9 (20.6–31.0)	56 (41–130)^*^^#^	106 (52–113)^*^^#^	52.4 (42.9–77.0)^*^^#^
*anteiso*-pentadecanoic (aC15:0)	*Staphylococcus, Bacillus, Corinebacterium*	377 (316–607)	307 (236–622)	325 (285–558)	411 (190– 469)	558 (367–822)
*anteiso*-heptadecanoic (aC17:0)	*Corinebacterium, Bacteroides, Micromonospora, Staphylococcus*	1601 (1215–2232)	1519 (967–2259)	1657 (1258–2068)	1879 (1105–1918)	2075 (1583–2589)
*anteiso*-nonadecanoic (aC19:0)	*Staphylococcus*	179 (136–213)	181 (139–236)	176 (160–206)	206 (158–212)	205 (196–269)
**CYCLOPROPYL FA**						
Cyclopropylheptadecanoic (C17cyc)	*Enterobacteriaceae, Pseudomonas, Alcaligenes*	1.60 (1.05–2.25)	1.8 (1.4–2.7)	3.4 (2.0–9.1)^*^^#^	5.6 (5.3–6.3)^*^^#^	4.20 (3.40–5.45)^*^^#^
Cyclopropylnonadecanoic (C19cyc)	*Lactobacillus, Enterococcus*	19.9 (13.05–32.7)	22.3 (18.5–23.1)	39.5 (25.3–136.2)^*^^#^	93.3 (45.2–112.4)^*^^#^	46.3 (37.5–56.4)^*^^#^
**UNSATURATED FA**						
*cis*-9-tetradecenoic (C14:1d9)	*Clostridium*	20.0 (7.5–28.8)	6.2 (5.2–11.5)^*^	12.2 (7.3–18.7)	11.2 (7.8–28.1)	29.6 (13.6–34.9)^#^
*cis*-11-tetradecenoic (C14:1d11)	*Nocardia*	142 (78–364)	67.8 (45.6–123.6)^*^	79 (61–127)^*^	167 (141–228)^#^	169 (97–186)
*cis*-9-pentadecenoic (C15:1d9)	*Clostridium propionicum, Bacteroides hypermegas*	8.30 (5.00–15.15)	4.5 (3.7–6.6)^*^	6.5 (5.3–11.7)	6.8 (5.6–8.0)	9.1 (7.7–23.8)^#^
*cis*-7-hexadecenoic (C16:1d7)	*Clostridium ramosum, C. innocuum, C. clostridioforme*	1054 (859–1290)	760 (623–877)^*^	847 (597–978)^*^	1058 (974–1195)^#^	846 (710–1019)
*cis*-11-hexadecenoic (C16:1d11)	*Ruminococcus, Nocardia, Fusobacterium*	157 (110–236)	89 (69–129)^*^	133 (101–169)	164 (139–189)^#^	193 (135–225)
*cis*-9-heptadecenoic (C17:1d9)	*Candida albicans*	210 (149–301)	248 (181–292)	421 (222–697)^*^	564 (414–589)^*^^#^	364 (271– 634)^*^
*cis*-11-octadecenoic (C18:1d11)	*Bacteroides, Fusobacterium, Enterobacteriaceae, Pseudomonas, Lactobacillus*	4594 (2655–5915)	3826 (3643–5929)	4533 (3283–5030)	5131 (4372–6121)	4060 (3743–4956)
*cis*-9-eicosenoic (C20:1d9)	*Propionibacterium jensenii, Actinomyces*	840 (725–954)	732 (631–846)	779 (665–841)^†^	855 (846–1044)^*^	973 (916–1044)^*^^#^
*cis*-11-eicosenoic (C20:1d11)	*P. jensenii, Actinomyces*, S*treptococcus*	72.2 (54.6–100.9)	64.2 (59.0–98.2)	61.3 (50.8–76.7)^†^	101 (60–116)	81.4 (78.8–93.4)
**ALDEHYDES**						
*cis*-vaccinic (C18:1d11A)	*Eubacterium moniliforme, S. mutans, C. ramosum*	4072 (3260–5252)	2809 (2067–5621)	3353 (3027–4578)	3631 (2101–4475)	3219 (2634–3289)^*^
Oleic (C18:1d9A)	*Bifidobacterium bifidum, Propionibacterium*	11518 (9277–13406)	11276 (8353–11640)	9469 (8788–12420)	9166 (8597–9278)	9110 (8020–10059)^*^
Anteisoheptadecanoic (aC17A)	*Eubacterium lentum*, *Propionibacterium acnes*	1014 (688–1474)	1279 (1097–1768)	1316 (988–1927)^*^^†^	788 (779–941)	789 (645–845)
*iso*-palmitic (iC16A)	*E. lentum*	386 (278–538)	511 (348–613)	465 (372–576)	352 (299–372)	355 (263–365)
Anteisopentadecanoic (aC15A)	*Eubacterium, Peptostreptococcus*	119 (75–184)	86.8 (56.6–142.8)	111.6 (90.5–151.4)	152 (83–182)	102 (95–148)
Myristic (C14A)	*E. lentum, B. bifidum*	85.3 (61.3–134.0)	99.5 (59.4–107.3)	96.4 (85.1–122.7)	88.3 (73.5–102.0)	136 (85–152)
*iso*-myristic (iC14A)		92.6 (57.4–131.3)	118 (81–151)	109 (93–119)^†^	66.6 (65.2–67.8)	71.1 (61.4–77.9)

a The majority of bacteria belongs to the four phyla found in the human gut (Firmicutes, Bacteroidetes, Actinobacteria, and Proteobacteria).

b Codes of chemical substances are given in parenthesis.

c Median values (ng/ml) followed by the interquartile range in parenthesis (25th and 75th percentiles).

d FMF patients without colchicine treatment.

e FMF patients on colchicine therapy.

Statistical significance of intergroup differences according to the Mann–Whitney U-test.

* p < 0.05 significance compared to healthy controls.

# p < 0.05 significance compared to the group of FMF patients in attack period without colchicine treatment.

† p < 0.05 significance compared to the group of colchicine-treated FMF patients in remission period.

Interestingly, concentrations of systemic microbial markers were similar between the colchicine-treated and untreated patients in remission, we found no statistically significant differences between them. At the same time, in the attack period, a statistically significant two-fold increase in the level of certain systemic microbial markers was found among the colchicine-treated patients compared to the untreated group. These long chain fatty acids (LCFAs) were: iC18:0 (marker present in *Peptostreptococcus*, *Clostridium difficile*, and *Bifidobacterium*), C17cyc (present in *Enterobacteriaceae*, *Pseudomonas*, and *Alcaligenes*), and C19cyc (present in *Lactobacillus* and *Enterococcus*).

In general, statistically significant pairwise differences in concentration of microbial markers were not associated with the use of colchicine, but rather with the health status or the disease stage (Table 1). In particular, the corresponding values were significantly different among the group of colchicine-treated patients in attack period and both groups of patients in remission compared to control. For example, significantly higher levels of systemic hydroxy FAs were revealed in colchicine-treated patients in attack compared to the normal subjects (with the exception of 3hC12:0). Even higher levels of almost all hydroxy FAs were uncovered in the remission stage (irrespectively of colchicine intake) compared to control (Table 1). It is noteworthy that 2hC12:0 and 3hC12:0 are the signature compounds of non-fermentative Gram-negative bacteria (NFB), while 3hC18:0 and 10hC18:0 are the reference markers for *Helicobacter pylori*, and *Clostridium perfringens*, respectively.

Interestingly, the concentrations of systemic hydroxy FAs such as i18, 17cyc, and 19cyc, but with the exception of 3hC12:0, were similar between the patients in attack without colchicine treatment and the normal subjects. Surprisingly, colchicine treatment in this group leads to the elevation of these microbial markers up to two- and four-fold compared to control (Table 1).

On the contrary, the concentration of another group of systemic microbial components, unsaturated FAs, was significantly different between the patients in attack period without colchicine treatment and controls (Table 1). In particular, the concentrations of C14:1d9, C14:1d11, C15:1d, C16:1d7, and C16:1d11 were significantly decreased in these patients compared to controls. These microbial components are relevant to *Clostridium*, *Nocardia*, *Clostridium propionicum*, *Bacteroides hypermegas, Ruminococcus*, *Clostridium ramosum*, and *Clostridium innocuum* (Table 1). The effect of colchicine treatment in these patients was limited and led to the reduction of only C14:1d11 and C16:1d7 compared to the healthy subjects. It should be noted that there were no statistically significant differences in the level of unsaturated FAs in the remission period vs. control, regardless of colchicine treatment.

Without the colchicine therapy, concentrations of FAs such as iC18:0, C17cyc and C19cyc in the metabolome of patients in remission were almost four-fold higher compared to the control and attack-period subjects (Table 1). In the case of the colchicine therapy, the concentration of these metabolites was very similar, irrespective of the disease stage, and were elevated only twice vs. control and patients in attack without colchicine treatment. The same tendency, but to a lesser extent, was observed for C17:1d9, one of the biomarkers of *Candida albicans*.

As for the disease stage, comparative analyses of microbial components in the systemic metabolomes of FMF patients during attack and remission periods revealed that these two groups are substantially more different without the treatment by colchicine, while the corresponding therapeutic intervention negates these differences (Table 1). In particular, in the absence of the colchicine therapy, the systemic concentrations of 3hC18:0, 10hC18:0, C17cyc, C19cyc, C14:1d11, C16:1d17, C16:1d11, and C17:1d9 were significantly reduced in the attack period compared to remission, while under the therapy these concentrations were very similar, with the exception of C14:1d11 (Table 1).

The negating effect of the colchicine therapy on the metabolome profile was not consistent through the range of all metabolites though. It may also contribute to the appearance of significant differences in the systemic microbial LCFAs between the attack and remission periods. This was noted for the following substances: C20:1d9 and C20:1d11 (biomarkers of *Propionibacterium jensenii*, *Actinomyces*, and S*treptococcus*), a17a (*Eubacterium lentum* and *Propionibacterium acnes*) and i14a (Table 1). The direction of the concentration change is also not consistent: the latter two aldehydes were elevated in the attack compared to remission period, while the others demonstrated the opposite trend.

The only component of the metabolome that was significantly elevated in remission vs. attack period, regardless of the colchicine therapy, was 3hC12:0 (biomarker of NFB) (Table 1). It was also significantly elevated compared to the healthy subjects.

**Figure 1:**
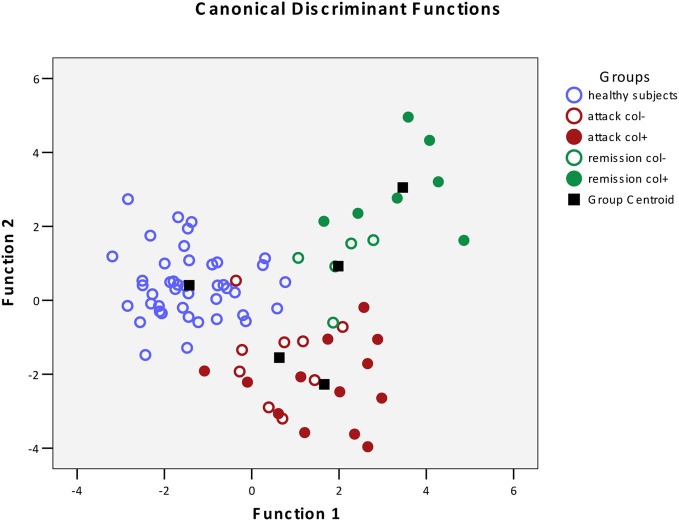
**Scatterplot of DA model based on systemic concentration of microbial components of the metabolome.** Number of variables in the model—30 and grouping consists of five groups. Root 1, 2—discriminant functions 1 and 2 (1st and 2nd canonical roots). 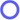, Healthy subjects; 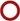, FMF patients in attack period without colchicine treatment; 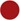, FMF patients in attack period under colchicine treatment; 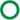, FMF patients in remission period without colchicine treatment; 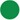, FMF patients in remission period under colchicine treatment; 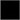, group centroid.

The results of the pairwise analyses do suggest that there are substantial differences in the metabolome due to the health status, stage of disease, and the colchicine therapy. Due to the complexity of these effects, further analyses included multivariate statistics, in particular, DA, to verify if the concentration of microbial products in the systemic metabolome may discriminate among the groups and serve as a predictor of a group membership. To test this approach, the raw data were subjected to DA, using 30 microbial products in the metabolome as predictors (Figure 1). According to this analysis, the variables accurately discriminated the groups and form five separate clusters corresponding to the cohorts under investigation. Moreover, the predicted group membership was also fairly accurate, with 90.9% of original grouped cases correctly classified (Table 2). The statistical significance of the DA model was also well supported by the Wilks's lambda test, with the corresponding coefficient value of 0.026 (Table 3).

**Table 2:** **Predicted group membership based on 30 variables**.

	**Groups**	**Predicted group membership^a^**					**Total**
		**1**	**2**	**3**	**4**	**5**	
Count	1	40	3	0	0	0	43
	2	1	7	1	0	0	9
	3	1	1	11	0	0	13
	4	0	0	0	5	0	5
	5	0	0	0	0	7	7
%	1	93.0	7.0	0.0	0.0	0.0	100.0
	2	11.1	77.8	11.1	0.0	0.0	100.0
	3	7.7	7.7	84.6	0.0	0.0	100.0
	4	0.0	0.0	0.0	100.0	0.0	100.0
	5	0.0	0.0	0.0	0.0	100.0	100.0

a 90.9% of original grouped cases are correctly classified.

**Table 3:** **Wilks's lambda test**.

**Test of function(s)**	**Wilks' lambda**	**Chi-square**	**Df**	**Sig.**
1–4	0.026	213,837	120	0.000
2–4	0.109	129,794	87	0.002
3–4	0.359	60,007	56	0.333
4	0.656	24,677	27	0.593

The DA analysis showed distinct clustering of both groups of patients in remission from other cohorts studied with 100% accuracy (Table 2). It should be noted that in the remission period separation of patients subjected or not subjected to colchicine treatment was observed with 100% accuracy, despite the fact that, as it was mentioned above, there were no statistically significant pairwise differences between the colchicine-treated and untreated patients at this stage of the disease. This demonstrates that the variables, i.e., the concentrations of microbial components in the metabolome, are highly cohort-specific.

Since colchicine is the main drug used to manage this disease, it was expected that the profiles of microbial products in patients under the therapy would be “normalized,” following the suppression of clinical manifestations of the disease. However, the effect of colchicine was quite the opposite, forming new distinct profiles of microbial products in the metabolome, well separated from the healthy controls and FMF patients without the therapy.

## Discussion

Previously, we have established that FMF results in a specific restructuring of gut microbiota affecting the major phyla present (Khachatryan et al., 2008). Correspondingly, the profile of microbial LCFAs circulating in the blood is also specific for the diseased and healthy states (Ktsoyan et al., 2010). In this work, we addressed the question whether the colchicine therapy that is used to suppress the clinical manifestations of FMF and to extend the remission periods could also have an effect on the level and profile of microbial LCFAs in systemic circulation. Surprisingly, the suppression of clinical signs of the disease by the therapy does not result in the normalization of microbial LCFAs in the metabolome. On the contrary, the treatment results in the formation of new distinct microbial LCFA profiles in the metabolome, which are well separated from the corresponding profiles of healthy controls and FMF patients without the therapy (Figure 1).

The effect of colchicine on gut microbiota can be exerted in two possible ways: (1) direct influence of this peroral drug on gut microbiota and (2) indirect influence through the host responses to the therapy. There are very few literature sources concerning the direct effect of colchicine toward microorganisms. The antimicrobial effect of colchicine was tested *in vitro* against a number of potential pathogens and the MICs obtained were well within the concentration range of the most common antibiotics used (Ozçelik et al., 2011). However, the therapeutic dosage of antibiotics prescribed is much higher, more than by the two orders of magnitude, than the usual therapeutic concentrations of colchicine. It is highly unlikely therefore, that colchicine may exert any antimicrobial activity at the concentrations used for FMF therapy and contribute to the observed shifts of microbial LCFAs in systemic circulation.

In the latter scenario, colchicine may interfere with the formation of microtubules (Massarotti et al., 2012), thereby affecting mitosis and other microtubule-dependent functions such as diapedesis. As a consequence of the reduced mobility, the infiltration of leukocytes into the affected sites may be impeded, thus reducing the subclinical inflammation in the gut (Niel and Scherrmann, 2006). The restoration of the compromised gut epithelial barrier due to the colchicine therapy may result in a lower frequency of microbial translocation in the gut. Indeed, when assessing the influence of colchicine treatment on systemic immunoglobulin isotypes in FMF patients, we found the “normalizing” effect of the therapy on the level of systemic IgAs that were directed toward the protein antigens of commensal gut bacteria (Manukyan et al., 2008b). The concentration of microbial LCFAs in the metabolome, however, is less responsive to the colchicine intake and, for example, there are no statistically significant differences between the colchicine-treated and untreated patients in the remission period. Moreover, in disease attacks, the level of certain microbial LCFAs such as iC18:0, C17cyc and C19cyc is actually significantly higher in the colchicine-treated cohort compared to the untreated (Table 1). This is surprising since the total number of fecal bacteria falls substantially during the attack periods (Khachatryan et al., 2008) and suggests the overgrowth of certain groups of bacteria with these specific LCFA signatures due to the colchicine treatment.

Despite the absence of pairwise statistically significant differences between the colchicine-treated and untreated cohorts in the remission period (Table 1), these groups are clearly separated when the multivariate statistics analysis tools are applied (Figure 1). The concentration of 30 microbial LCFAs in the metabolome used as variables in the DA model allows predicting the disease stage and the impact of the therapeutic intervention with a 100% accuracy (Table 2). Although the vast majority of microbial markers in the attack period are also not responsive to the treatment (Table 1), the two groups form distinct clusters in the DA model (Figure 1), albeit with a less predictive membership accuracy compared to the remission groups (Table 2). These results suggest that the colchicine therapy, which suppresses the clinical signs of the disease and extend the asymptomatic remission periods, does not “normalize” the gut microbiota composition as judged by the profiles of microbial LCFAs in the metabolome. Moreover, the therapy actually shifts the composition toward a new equilibrium, which is different from the healthy and untreated disease states. This new equilibrium is certainly mediated by the host, which responses are modified by the drug action.

The colchicine therapy restricts the infiltration of neutrophils to the affected sites due to the interference with the microtubule-dependent functions such as diapedesis (Massarotti et al., 2012). Another effect of colchicine is realized at the transcription regulation level and includes inflammation and interferon-associated genes (Ben-Chetrit et al., 2006). We hypothesize that as a consequence of the limited neutrophils infiltration and differential expression of inflammation and interferon-associated genes during the colchicine treatment, the balance of pro- and anti-inflammatory mediators is shifted, similarly to the shifts in cytokine profile that are disease-specific (Manukyan et al., 2010). This may result in differential immune responses in colchicine-treated and untreated FMF patients. Indeed, in our preliminary work, we found the increased phagocytic activity of neutrophils in the blood of colchicine-free FMF patients compared to the treated at the stage of remission and healthy controls, up to two- and four-fold, respectively (unpublished data). Moreover, it appears to be that colchicine, in addition to its well-known anti-inflammatory activity, may actually exert a potent pro-inflammatory activity up-regulating, in particular, the expression of IL-1β and IL-8 mRNAs (unpublished data). As a result of the restructured inflammatory environment in the gut, the microbiota composition is also altered with the concomitant alteration in the concentration of microbial LCFAs entering into the systemic circulation. Further investigations are needed to validate this hypothesis regarding the effects of colchicine therapy in FMF, with larger cohorts and parallel microbial diversity analyses.

### Conflict of interest statement

The authors declare that the research was conducted in the absence of any commercial or financial relationships that could be construed as a potential conflict of interest.
